# Handheld Near-Infrared Fluorescence Imaging Device Using Modified Action Cameras for Peri-Operative Guidance of Microvascular Flap Surgery

**DOI:** 10.3390/jcm10030410

**Published:** 2021-01-21

**Authors:** Hyunwoo Yang, Jihong Kim, Woong Nam, Hyung Jun Kim, In-ho Cha, Dongwook Kim

**Affiliations:** Department of Oral & Maxillofacial Surgery, Yonsei University College of Dentistry, 50-1 Yonsei-ro, Seodaemun-gu, Seoul 03722, Korea; baachooo@yuhs.ac (H.Y.); Curry30@yuhs.ac (J.K.); OMSNAM@yuhs.ac (W.N.); KIMOMS@yuhs.ac (H.J.K.); CHA8764@yuhs.ac (I.-h.C.)

**Keywords:** ICG, ICG-NIRF, indocyanine green, flap monitoring, microvascular surgery

## Abstract

Indocyanine green near-infrared fluorescence (ICG-NIRF) imaging has recently come into use as a novel method in peri-operative microvascular flap assessment. However, a majority of the many commercial devices launched for clinical use lack mobility, portability, and cost-efficiency and are thus unsuitable for intra-oral applications. This study introduces a cost-effective, customized, handheld NIRF device following principles of ICG-NIRF imaging. Moreover, the novel characteristics of our prototype, considered in conjunction with a literature review highlighting the significance of fluorescence devices in microvascular surgery, point to a new generation of devices for use in microvascular flap surgery.

## 1. Introduction

Despite dramatically improved survival rates, microvascular flap loss is sometimes an inevitable and potentially costly disaster [1,2,3]. Among many factors affecting free-flap survival and prognosis, proper flap monitoring and precisely locating perforators and their feeder vessels are of the utmost importance [2]. When monitoring transferred flaps, the prompt detection of flap compromise and immediate surgical intervention, such as vessel re-anastomosis, are also critical because they are strongly related to a high flap salvage rate [4]. Accordingly, most surgeons emphasize a routine flap monitoring protocol to maximize recognition of early flap compromise. The critical period of post-operative flap monitoring is generally the 48 to 72 h immediately after surgery. As in our department, in-house staff and nurses abroad are generally involved in following flap monitoring protocols, with a frequency as high as every hour (q1h) [5]. Heterogeneous groups of observers over time, however, sometimes render erroneous, non-objective flap evaluations. For this reason, microvascular surgeons have long sought an objective and intuitive flap surveillance system suitable for a majority of observers.

Various devices have been proposed for an optimal peri-operative flap monitoring system. Systems can be broadly categorized as invasive and non-invasive. Implantable Doppler/venous couplers, oxygen tension monitoring, tissue pH monitoring, microdialysis, Technetium-99m scintigraphy, and contrast-enhanced Doppler are invasive while acoustic, color, laser Doppler, microlight-guided spectrophotometry, surface temperature monitoring, and tissue oximetry are non-invasive [6]. However, no single method has been universally adopted due to one or more shortcomings with regard to ease of use, invasiveness, sensitivity, and cost. Most importantly, devices are operator-dependent in terms of both skill and prior anatomical knowledge of the transferred flap [7].

Fluorescence angiography with indocyanine green (ICG) has emerged as a new method for evaluating tissue perfusion [8]. This real-time vascular imaging technique, which has been applied in many fields of clinical medicine for various purposes, can significantly facilitate the mapping of perforators and the iterative confirmation of anastomosed vessel patency [9] as well as tissue perfusion. Unique characteristics of ICG fluorescence imaging fulfill many of the ideal flap-monitoring method criteria set forth by Chao [10].

However, various medical ICG devices on the market are neither cost-effective nor suitable for intra-oral monitoring. In most cases, they lack portability and mobility, nullifying the unique advantages of ICG fluorescence imaging in terms of oral and maxillofacial reconstruction. Considering the basic principles and structures of ICG imaging devices, we devised a handheld ICG prototype with action cameras for peri-operative use in microvascular surgery.

This study presents a novel type of handheld ICG prototype specially designed for oral and maxillofacial microvascular flap reconstruction and includes a comparative analysis with commercially available devices. We also conducted a retrospective preliminary study based on a microvascular flap patient cohort that suggests the potential of our device for use in ICG-based flap surgery.

## 2. Materials and Methods

### 2.1. Designing an ICG-NIRF Prototype Incorporating Modified Action Cameras

**Figure 1:**
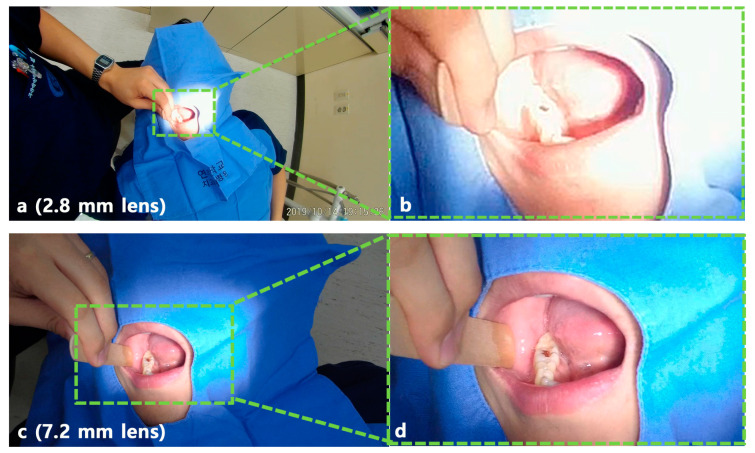
Snapshot of recorded video. (**a**) Image of action camera with original lens, (**b**) magnified photo of (**a**), (**c**) image with customized action camera, (**d**) magnified photo of (**c**).

This study used the SJCAM Pro Action Camera (SJCAM, Shenzhen, China) (Table 1) due to its light weight, 4k resolution, image stabilization, and Wi-Fi connection. The battery provides up to 90 min of wireless recording. However, its 2.8 mm wide-angle lens renders the acquired images unsuitable for oral surgery (Figure 1a,b). To make the device more usable for oral surgery, we replaced the 2.8 mm stock lens with a 7.2 mm telephoto lens for a substantial improvement in the acquired image (Figure 1c,d).

**Figure 2:**
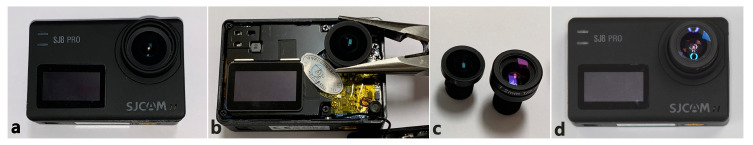
Modified action camera with 7.2 mm lens. (**a**) SJCAM Pro Action Camera (SJCAM, Shenzhen, China), (**b**) Removing the front lid of action camera and 2.8 mm stock lens, (**c**) 2.8 mm stock lens and 7.2 mm telephoto lens, (**d**) modified action camera with 7.2 mm telephoto lens.

After removing the front lid of the action camera, the 2.8 mm stock lens was replaced with a 7.2 mm telephoto lens (Figure 2).

**Figure 3:**
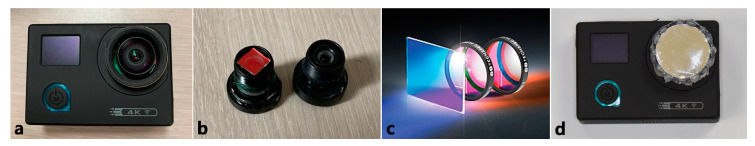
Modified action camera with 7.2 mm lens and band-pass filter. (**a**) SJCAM Pro Action Camera (SJCAM, Shenzhen, China), (**b**) Stock lens with IR filter(left), 7.2 telephoto lens(right), (**c**) Band-pass filter, (**d**) modifed action camera with 7.2 telephoto lens and band-pass filter.

**Figure 4:**
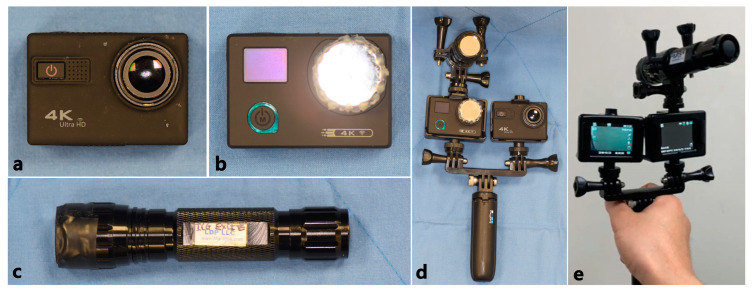
ICG-NIRF prototype and its components. (**a**) Modified action camera with 7.2 mm lens, (**b**) modified action camera with 7.2 mm lens and band-pass filter, (**c**) modified LED light with band-pass filter, (**d**) front of ICG-NIRF prototype, (**e**) back of ICG-NIRF prototype.

The ICG-NIRF (indocyanine green near-infrared fluorescence) equipment consists of an LED light source of a specific wavelength (760–805 nm), which excites the ICG, and a sensor to detect the specific wavelength (800–845 nm) of fluorescence [11]. The light source of our prototype device was fabricated by adding a band-pass filter to an LED flashlight (XNiteFlashICG, Maxmax.com, Carlstadt, NJ, US.) so that it transmitted at a wavelength of 780 nm. The sensor was made by modifying an action camera’s IR filter with a band-pass filter (Figure 3c,d), which transmits light in the 830 nm region, and a 7.2 mm telephoto lens. Finally, the NIR light source, POV (point of view) recording action camera, and ICG-NIR action camera were combined into a single hand-held device on a GoPro mount (Shorty Mount Adapter, GoPro, SM, USA) (Figure 4).

### 2.2. Evaluation of Device Resolution in Terms of Pixel Intensity

**Figure 5:**
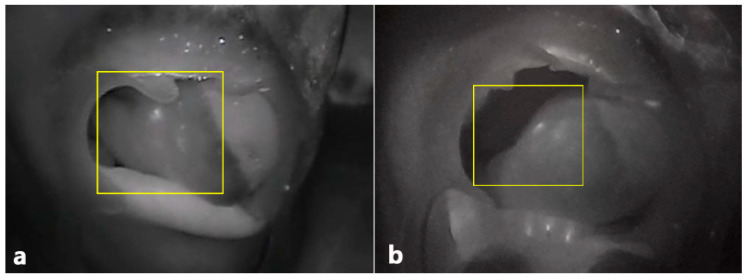
ICG-NIRF image of transferred flap. (**a**) Post-operative ICG-NIRF imaging with Fluobeam^®^, (**b**) post-operative ICG-NIRF imaging with the prototype.

The performance of our ICG-NIRF imaging device was evaluated in terms of pixel intensity (gray value) as extracted from a line between two points within a region of interest (ROI: fluorescent area of transferred flap) using images captured by our device and the Fluobeam^®^ device (Fluoptics, Grenoble, France) (Figure 5). We visualized the performance of each device on a two-dimensional graph in which the signal intensity (gray value) is set on the vertical axis and the distance in pixels is set on the horizontal axis.

### 2.3. Preliminary Retrospective Cohort Study of Microvascular Flap Patients.

The data of all patients who underwent microvascular flap surgery between January 2018 and December 2020 were reviewed in this retrospective study, with the exception of those under 18 years of age and those with unobservable flaps such as a buried flap. All operations were performed by the same microvascular team in our department. A total of 61 patients were enrolled and the flaps of 7 patients were intra-operatively designed and post-operatively monitored utilizing the ICG-NIRF prototype. ICG was injected intravenously (0.5 mg/kg body weight) into a peripheral vein prior to the skin pedicle design and within 72 h after microvascular anastomosis.

The study was approved by the Ethics Review Board of the Yonsei University Dental Hospital Institutional Review Board (IRB No.2-2020-0079). Written or verbal informed consent was not obtained from any participants because the IRB waived the need for individual informed consent as the design of this retrospective study was non-interventional and all data were analyzed anonymously.

Observational categories included demographic characteristics (gender and age) of enrolled patients, type of microvascular flap, operation time, peri-operative usage of the ICG device, presence of post-operative complications, flap necrosis, and pathology of the primary lesion.

The statistical analysis was performed using IBM SPSS 26.0 software. Baseline differences between the ICG group and non-ICG group were assessed using an independent *t* test for continuous variables and a Fisher’s exact test for categorical variables. To evaluate the impact of the ICG device on independent variables, a univariate logistic regression analysis was done as well. A *p* < 0.05 was considered significant.

## 3. Results

### 3.1. Clinical Evaluation

**Figure 6:**
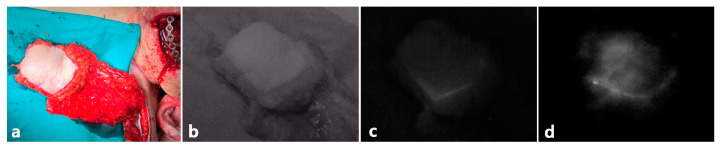
Time difference ICG perfusion of pectoralis major myocutaneous (PMMC) flap. (**a**) Original image of pectoralis major myocutaneous (PMMC) flap, (**b**) before ICG injection, (**c**) 1 min after ICG IV injection, (**d**) 2 min after ICG IV injection.

**Figure 7:**
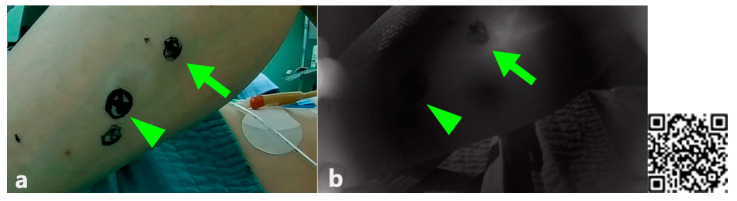
Different outcome in locating perforators between two devices(Doppler and ICG-NIRF prototype). (**a**) Locating perforators with Doppler, (**b**) locating perforators with a DIY ICG-NIR prototype (note that both arrow and arrowhead areas were positive on Doppler signal; however, only the site with the arrow was fluorescent), (**c**) QR code for ICG, free flap, and post-operative flap monitoring.

**Figure 8:**
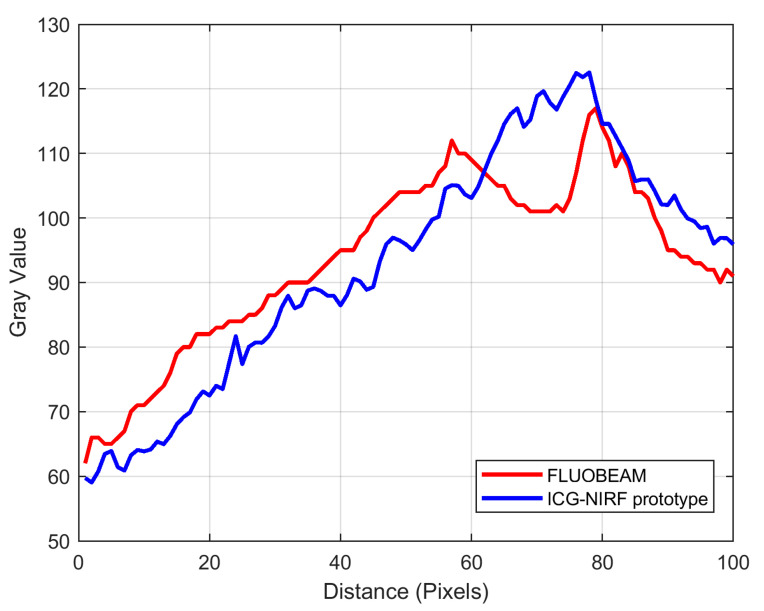
Two-dimensional graph of pixel intensity in the ICG fluorescent area of transferred flap.

The indocyanine green imaging prototype device revealed the vascular network of the raised flap, perforator location, and anastomosed vessel patency while enabling post-operative flap monitoring (Figure 6 and Figure 7). Moreover, in contrast to the hand-held Doppler device, our prototype managed to detect a false positive perforator position (Figure 7). Comparing similar waveforms obtained from Fluobeam^®^ with those of our prototype in the range of 60–80 distance (pixels), we found that our device achieved a higher maximum value of pixel intensity (Figure 8).

### 3.2. Statistical Evaluation

There were seven patients in the ICG group and 53 patients in the non-ICG group. There were no significant differences in the demographic characteristics (gender and age), operative time, flap type, incidence of postoperative complications, and flap necrosis (Table 2). Backward stepwise logistic regression analysis revealed that the ICG device did not influence operation time, incidence of complication, or flap necrosis in this study (Table 3).

## 4. Discussion

For the past few years, various NIRF (near-infrared fluorescence) imaging systems have been developed and clinically validated for ICG angiography in the peri-operative assessment of flap viability [12,13]. These systems visualize acquired images on an additional screen [11] or by in situ projection of NIRF images [14]. However, the screen-display method requires an additional screening tool, which may become an encumbrance in a space-limited operating room and could distract the surgeons, who must switch their field of view between the screen and the surgical site. The “in situ” projection method corresponds to clinicians’ natural visual perceptions. However, previously reported devices are bulky and require manual calibration at the specific working distance for co-registration between fluorescence emission and projective imaging [15]. In contrast, using our device is straightforward, as it not only allows natural visual perception but is miniaturized for handheld use.

It is crucial that fluorescence imaging instruments display both the visible and fluorescence images on the same screen [16]. This is especially important in microvascular surgery because the fluorescence image alone may reveal only a few significant details. Surgeons require the merged images to clearly perceive visible landmarks that help map the fluorescing areas within complex anatomical structures. Traditionally, two images are aligned through “image registration.” However, this involves complicated image processing. To avoid such a cumbersome procedure, some engineers utilize an ordinary beam splitter, a dichroic mirror that splits the beam into two parts: one for the visible camera and the other for the NIR camera. In this way, both cameras capture the same field of view (FOV), and no registration is needed provided the cameras have identical optics and are correctly aligned with each other. Throughout their research, the authors of the present study could confirm that many commercially available devices and newly devised prototypes require an additional screen display, which means they lack in situ projection [17,18,19]. However, the device presented here combines images from two separate action cameras: one camera’s embedded band-pass filter provides the fluorescence image, highlighting the perforator vessels and angiosomes, while the other action camera allows the operator to perceive color images.

A previous study introduced the action camera as a useful tool for recording first-person point-of-view surgical videos [20]. However, the wide-angle stock lens of an action camera is unsuitable for oral and maxillofacial surgery, which frequently requires intra-oral inspection and monitoring. Throughout several trials, the authors confirmed that a 7.2 mm telephoto lens provides optimal intra-oral projection (Figure 1), which is especially useful for monitoring flaps transferred to reconstruct intra-oral defects. As flaps transferred in narrow oral cavities are small and the glistening portions of ICG fluorescence may be even smaller, a telephoto lens is indispensable for focusing on fluorescent regions within the oral cavity. Although neither an intra-operative nor post-operative ICG microvascular flap assessment requires much time, the authors confirmed that their prototypes continuously displayed the images for 90 min without an external battery and over 6 h with the aid of an external battery. Most importantly, the modified action-camera device was light, cost-effective and provided higher quality-resolution compared to other POV devices.

As mentioned, ICG-NIRF devices operate on a relatively simple principle, utilizing a light source of a specific wavelength to excite ICG, a sensor to detect the specific fluorescence wavelength (800–845 nm), and two filters, one that allows transmission of a certain wavelength from the light source and another that effectively blocks all undesired wavelengths from reaching the sensor.

However, most commercially available ICG-NIRF devices are expensive despite their relatively ordinary components (Table 4 and Table 5).

The compromised free flap salvage rate is inversely related to the delay between the onset of ischemia and its clinical assessment [21]. Unfortunately, clinical symptoms may be insignificant early on and often noticed only after irreversible flap compromise [22]. Some might question the need for an ICG device given the portable Doppler devices traditionally used to identify perforators and monitor the post-operative status of transferred flaps as well as conduct clinical examinations. However, clinicians and researchers have noted that while a handheld Doppler can indicate the direction of blood flow, it fails to visualize the size of the vessel or its flow volume, sometimes resulting in false-positive or false-negative results [7]. As seen in Figure 7, two septocutaneous perforators were marked via a Doppler device prior to the raising of the flaps. However, the confirmation process by the ICG-NIRF revealed that the distal perforator was the false positive. If the cutaneous flap had been designed without the proximal perforator or a visual confirmation, partial or total flap necrosis would have been inevitable. Although a color Doppler can obviate some risks, most Doppler devices are clearly examiner-dependent and require prior anatomical knowledge of skin perforator locations. Moreover, the handheld Doppler is frequently not specific for the vessel of interest. The examiner may be deceived by an adjacent major vessel releasing a louder signal. Sometimes it is impossible to detect a cutaneous signal from a well-perfused flap. Either situation can lead to erroneous conclusions about the health of the flap [23]. In his study, Lohman et al. confirmed that among many examining methods, the Doppler signal never provided the initial sign of pending microvascular complications. In one of his patients, the external Doppler signal ceased 2.3 h after a traction injury to the anastomoses [24].

Frequent post-operative clinical examinations to assess skin color, flap temperature, and capillary refill time are obligatory but extremely laborious, and interpreting the findings requires experience. Moreover, even a clinician with sufficient skill in assessing a potentially compromised flap may have too many other clinical responsibilities to be able to recognize early signs of failing flaps. Furthermore, different observers may reach different conclusions depending on their respective degrees of expertise [25]. The dilemma reaches its limit when a flap appears healthy and the signal from the Doppler is poor whether due to the shortcomings of the Doppler devices mentioned above or an impending flap compromise. For these reasons, the combination of the Doppler device and a physical examination alone is not always reliable, necessitating objective, observer-independent methods [24]. During post-operative monitoring hours, residents on duty managed to detect complications in Doppler-positive flaps that showed significantly decreased fluorescence compared to the intra-operative record. Immediate salvage re-anastomosis prevented one flap necrosis.

Various studies support the intuitive and objective aspects of the ICG-NIRF, which directly indicates flap perfusion through the intensity of ICG fluorescence [26]. Many compromised flaps with inconspicuous clinical symptoms were detected by ICG-NIRF application prior to those in our study. Bigdeli et al. diagnosed venous congestion of an ALT (antero-lateral thigh) flap at an early stage and eliminated a thrombus via re-anastomosis [27]. In 2009, Holm et al. applied the ICG-NIRF to assess anastomotic patency in 50 consecutive free flaps. Interestingly, the surgeon’s decision not to augment vessel patency via anastomosis intraoperatively, despite the occlusion detected by ICG angiography, always resulted in flap loss or the need for early flap re-exploration [26].

The ICG-NIRF also crucially enables sophisticated flap tailoring. In obese patients with DIEP (deep inferior epigastric perforator) flaps, in which the angiosome or perforasome within the fat layer is unpredictable, flap tailoring following the zonal classification of Hartramp or Holm may be partially or totally imprecise [28,29]. The ICG-NIRF facilitates an intuitive understanding of complex surgical perfusion scenarios, aiding surgical decisions by providing an augmented reality view of perfusion. Therefore, ICG angiography enables individual flap planning by excluding the region of malperfusion, critically reducing the rate of partial flap necrosis or fat necrosis.

Like other screening tools, the ICG-NIRF method has limitations. As it requires an externally observable perfused surface, the technique cannot map buried flaps, such as in pure muscle [30]. Additionally, unlike continuous flap monitoring techniques such as laser Doppler flowmetry, tissue oxygen tension measurement, and tissue pH-metry, the ICG-NIRF only illuminates the area of flap perfusion in a particular time range [31,32]. However, given that the ICG is nontoxic and rapidly binds to plasma proteins, repeated applications are possible [33,34]. Furthermore, some authors have noted the excessive sensitivity of the ICG-NIRF. Self-limiting vascular vasospasms and a subsequently prolonged ICG-uptake and clearance may confuse the clinicians assessing a compromised flap [30]. This points to the need for correlation with clinical examination.

Despite our hands-on experience of the prototype’s versatile peri-operative utility, the significance of our device is statistically unconfirmed. This may be due to the retrospective nature of the study and limited size of the patient cohort. Including a larger number of patient cohort along with a systemized flap evaluating protocol may further establish the clinical validity of the ICG-NIRF device.

## 5. Conclusions

A cost-effective ICG-NIRF device was designed and prototyped following basic principles of ICG-NIRF imaging. Considering the benefits of ICG-NIRF, our newly devised prototype holds promise as a contribution to oral and maxillofacial surgery, especially in the field of microvascular flap surgery. It can especially be recommended for large institutions where an objective flap monitoring device is required to confirm clinical assessment. Dramatic improvements in mobility, portability, expense, and many other considerations addressed by our prototype constitute grounds for manufacturing a novel medical device. Bedside application of this device is expected to lead to improved microvascular flap quality and decreased morbidity. Furthermore, situations such as treating patients under home medical care due to peripheral vascular diseases or where the use of bulky medical devices is restricted will have a need for our miniaturized prototype. The approach here holds implications for clinicians working in countries where high-end equipment is unobtainable; we need to ask what other expensive devices can be made redundant through ingenuity and tinkering. However, this study needs further validation based on prospective study design with a larger patient cohort. In addition, a more ergonomic design and the ability to quantify fluorescence intensity remain to be pursued.

## Figures and Tables

**Table 1:** Action camera specification.

Item Specification	
Model name: SJ8 pro	Storage: micro-SD (Up to 128 GB)
CMOS sensor: SONY IMX377	Gyro stabilization: Yes (inner three-axis gyroscope)
Viewing angle: 170°	Image stabilization: Electronic image stabilization
Focal length: 2.8 mm	Battery Capacity: 1200 mAh
Wi-Fi: 2.4 G/5 G	Dimension: 28.8 × 62.5 × 41 mm
Sensor size: 1/2.3 inches	Weight (grams): 85
Sensor Resolution: 12 MP	Video output: 4K (3840 × 2160) 25/30/50/60 fps
Maximum aperture: F2.8	Video format: MP4

**Table 2:** Baseline characteristics and operational indexes of the patients in both groups.

	Non-ICG Group	ICG Group	*p*-Value
	(*N* = 53)	(*N* = 7)	
**Gender** (*n*,%)			0.349 ^a^
Male	29 (90.6%)	3 (9.4%)	
Female	24 (85.7%)	4 (14.3%)	
**Age** (mean ± SD)	54.8 ± 12.7	61.9 ± 13.2	0.171 ^b^
**Flap type**			1.244 ^a^
ALT	11 (84.6%)	2 (15.4%)	
FFF	19 (90.5%)	2 (9.5%)	
LDFF	1 (100.0%)	0 (0.0%)	
RFFF	22 (88.5%)	3 (11.5%)	
**Operation time** (mean ± SD)	10.47 ± 2.33	10.07 ± 2.28	0.429 ^b^
**Flap complication** (*n*,%)			2.097 ^a^
No	43 (91.5%)	4 (8.5%)	
Yes	10 (76.9%)	3 (23.1%)	
**Flap necrosis** (*n*,%)			0.856 ^a^
No	45 (90.2%)	5 (9.8%)	
Yes	8 (80.0%)	2 (20.0%)	

*ICG*—indocyanine green, *SD* standard deviation, *ALT*—antero-lateral thigh, FFF—fibular free flap, LDFF—latissimus dorsi free flap, RFFF—radial forearm free flap, ^a^ Fisher’s exact test, ^b^ independent *t* test.

**Table 3:** Logistic regression analyzing impact of ICG device on operation time, incidence of complication and flap necrosis.

Variable	ICG Use	
	OR (95% CI)	*p*-Value
Operation time	0.977 (0.638, 1.494)	0.913
Flap complication	4.229 (0.338, 52.894)	0.263
Flap necrosis	1.103 (0.069, 17.707)	0.945

OR—odds ratio, CI—confidence interval.

**Table 4:** Comparison of ICG-NIR devices.

Device	Excitation Source	Fluorescence Collection	Detector	Working Distance	Field of View	Depth of Penetration	Integration Time or Frame per Sec (FPS)
Photodynamic Eye (PDE), Hamamatsu	LEDs centered at 760 nm	Band-pass filter > 820 nm	CCD	20 cm	Not given, but limited	2 cm	Not specified
SPY, Novadaq	Laser emitting at 860 nm, 2.0–2.7 W	835 nm, “camera” not specified	CCD	30 cm	56 cm^2^	1 mm DOP	30 fps
Fluobeam 800, Fluoptics	Laser emitting at 780 nm, 7 mW	>820 nm, “camera”	CCD	20 cm	130 cm^2^	2.5 cm	25 images/sec
DIY ICG-NIR prototype	LED light sources with filter	Band-pass filter	CCD	45 cm	Variable	Not specified	Various

**Table 5:** Comparison of commercial device and ICG-NIRF prototype features.

	Commercial Devices	DIY ICG-NIR Prototype
Price	Over $70,000	$500
Image quality	Good	Acceptable
Size	Large	Small, handheld
Detector	CCD	
Working distance	20–30 cm	
Bedside monitoring	Unwieldy	Easy
Quantitative data	o	x
Free flap monitoring	o	
Perforator locating	o	

O : possible, X : impossible.

## Data Availability

The data that support the findings of this study are available from the corresponding author, upon reasonable request.
